# Distinct prognosis of biliary tract cancer according to tumor location, stage, and treatment: a population-based study

**DOI:** 10.1038/s41598-022-13605-3

**Published:** 2022-06-17

**Authors:** Mee Joo Kang, Jiwon Lim, Sung-Sik Han, Hyeong Min Park, Sun-Whe Kim, Woo Jin Lee, Sang Myung Woo, Tae Hyun Kim, Young-Joo Won, Sang-Jae Park

**Affiliations:** 1 Center for Liver and Pancreatobiliary Cancer, National Cancer Center, 323 Ilsan-ro, Ilsandong-gu, Goyang-si, Gyeonggi-do 10408 Republic of Korea; 2 Division of Cancer Registration and Surveillance, National Cancer Center, 323 Ilsan-ro, Ilsandong-gu, Goyang-Si, Gyeonggi-do 10408 Republic of Korea; 3 Department of Cancer Control and Population Health, Graduate School of Cancer Science and Policy, National Cancer Center, 323 Ilsan-ro, Ilsandong-gu, Goyang-si, Gyeonggi-do 10408 Republic of Korea; 4 Division of Health Administration, Yonsei University, Wonju, Republic of Korea

**Keywords:** Medical research, Oncology

## Abstract

Biliary tract cancer (BTC) has been inconsistently identified according to its location in epidemiological and clinical studies. This study retrospectively reviewed the treatment pattern and prognosis of BTC according to tumor location using the Korea Central Cancer Registry data of 97,676 patients with BTC from 2006 to 2017. The proportion of localized and regional Surveillance, Epidemiology, and End Results (SEER) stage was the highest in ampulla of Vater (AoV, 78.2%) cancer, followed by extrahepatic bile duct (BD, 68.3%), gallbladder (GB, 52.6%), and intrahepatic BD (49.5%) cancers. Overall, the “no active anti-cancer treatment” rate was the highest in intrahepatic BD (52.8%), followed by extrahepatic BD (49.5%), GB (39.6%), and AoV cancers (28.9%). The 5-year relative survival rate was the highest in AoV (48.5%), followed by GB (28.5%), extrahepatic BD (19.9%), and intrahepatic BD (10.8%) cancers, which significantly improved over time, except for intrahepatic BD cancer. In the localized and regional stage, older patients had a higher risk of receiving no active anti-cancer treatment in each tumor location after adjusting for period and sex. BTC statistics should be reported separately according to tumor location due to its distinct SEER stage distribution, treatment pattern, and prognosis. Care should be taken in elderly patients to reduce the rate of no active anti-cancer treatment.

## Introduction

Biliary tract cancer (BTC) includes a wide spectrum of diseases, including intrahepatic, perihilar, distal bile duct, ampulla of Vater, and gallbladder cancers^1^. Recently recognized globally increasing trends of intra- and extrahepatic bile duct cancer incidence emphasized the need for a thorough investigation of the risk factors of BTC^2–4^. Nevertheless, BTC has not been analyzed in a uniform manner; studies have either pooled the diseases into a single entity or selectively reported a few of the subgroups. Notably, BTC has various subgroups with distinct epidemiological and clinical features according to tumor location^1^. For example, the GLOBOCAN provides summary data of gallbladder cancer but none for other subgroups of BTC or BTC as a whole^5^. In Korea, the National Cancer Statistics and Death Statistics reported the pooled incidence and mortality rates of BTC (C23–C24), although the country has the highest incidence of BTC worldwide^6,7^. Intrahepatic bile duct cancer (C22.1) is included in the category of liver cancer (C22) under the current scheme of International Classification of Diseases (ICD-10)^8^; however, it was inconsistently included in the summary statistics of BTC. In addition, there has been a debate on the misclassification of perihilar bile duct cancer as intrahepatic bile duct cancer, which has been proven to have a significant impact on the incidence of intra- and extrahepatic bile duct cancer in a country where the disease is widespread^9–12^.

Consequently, the fragmented format of the current datasets has created limitations to obtaining a comprehensive overview of BTC in a systematic manner. It is crucial to understand the distinct epidemiology and prognosis of each anatomical location for proper decision-making regarding the treatment strategy for BTC^13,14^. Moreover, nationwide treatment patterns should be investigated to identify a subgroup of patients who are not part of the hospital-based treatment outcomes study^15^. In this study, epidemiological and clinical features including the treatment pattern and prognosis of BTC were investigated according to the tumor location through the use of a national population-based cancer registry data in Korea from 2006 to 2017.

## Materials and methods

### Data source

Epidemiologic data were obtained from the Korea Central Cancer Registry (KCCR), which has annually collected incidence data from the entire Korean population in all regions since 1999^12^. In the KCCR, the Surveillance, Epidemiology, and End Results (SEER) stage has been formally recorded since 2006; therefore, this study analyzed data from 2006 to 2017 to stratify treatment patterns and prognoses in relation to the SEER stage. The SEER stage defines localized stage as a malignancy limited to the organ of origin and regional stage as tumor extension beyond the limits of the organ of origin, by direct extension and/or regional lymph node(s) involvement^16^. For each tumor location included in this study, the localized stage was defined as follows: confined to the gallbladder: superficial invasion to the submucosa at the deepest layer (gallbladder [GB]); confined to the intrahepatic bile duct: solitary or multiple tumors with or without intrahepatic vascular invasion (intrahepatic bile duct [BD]); confined to the cystic duct, distal bile duct, extrahepatic bile duct, or perihilar bile duct: superficial invasion to the submucosa at the deepest layer (extrahepatic BD); confined to the ampulla, duodenal submucosa, sphincter of Oddi: perisphincteric invasion (ampulla of Vater [AoV])^16^.

### Patient cohort

The patient cohort was categorized into four subgroups according to tumor location based on the International Classification of Diseases, tenth revision (ICD-10)^8^, as follows: GB (C23, n = 27,324), intrahepatic BD (C22.1 excluding M8162/3, n = 29,328), extrahepatic BD (C24.0 including all M8162/3, n = 32,572), and AoV (C24.1, n = 8452). All BD cancer registered with a morphology code of “Klatskin tumor (M8162/3)” was reclassified as extrahepatic BD cancer, regardless of the registered topography^12^.

Demographic characteristics, incidence trends, changes in SEER stage distribution and treatment patterns, and survival outcomes were analyzed for all 97,676 BTC patients as a whole and for each tumor location. For a subgroup analysis of treatment patterns in relation to the SEER stage, 15,509 patients with unidentified SEER stage or treatment information were excluded (detailed number of each tumor location is described in each sub-section).

### First course of treatment

In the registry, the “First course of treatment” was recorded based on the documented cancer-directed treatment that was actually administered to the patients before disease progression or recurrence, within the first four months after the initial diagnosis^17,18^. As described in our prior report, nine categories of first course of treatment were aggregated into four groups: surgical first course of treatment (surgery alone, surgery with chemotherapy, surgery with radiotherapy, surgery with chemotherapy and radiotherapy); non-surgical first course of treatment (chemotherapy alone, chemotherapy with radiotherapy, and radiotherapy alone); no active anti-cancer treatment; and unknown^15^.

### Statistical analysis

The incidence rates are expressed as crude rate (CR) and age-standardized rate (ASR) per 100,000 individuals. The CR was calculated as the total number of cases divided by the mid-year population of the specified years. The ASRs per year were calculated using Segi’s world standard population^19^. The study period was divided into periods I (2006–2011) and II (2012–2017). Age was stratified into four groups (< 60, 60–69, 70–79, and ≥ 80 years) for the statistical tests. Adjusted risk ratios were analyzed using the binreg command in STATA version 16.1 (StataCorp LLC, TX, USA). Relative survival rates were estimated using the Ederer II method^20^ with some minor modifications, based on an algorithm written in SAS provided by Paul Dickman^21^. Analyses of survival were performed using SAS 9.4 (SAS Institute, Inc., Cary, NC, USA). All statistical tests were two-tailed, and the results were considered statistically significant at p < 0.05.

## Results

### Epidemiologic overview of biliary tract cancer

Among the 97,676 patients with BTC, 53.1% were aged 70 years or older. The male-to-female ratio was the lowest in GB cancer (0.76:1) and the highest in intrahepatic BD cancer (1.62:1; Table 1).

**Table 1:** Characteristics of patients with biliary tract cancer according to the tumor location.

Characteristics	Gallbladder (n = 27,324)	Intrahepatic bile duct (n = 29,328)	Extrahepatic bile duct (n = 32,572)	Ampulla of Vater (n = 8452)	All biliary tract cancer (n = 97,676)
**Age at diagnosis**					
00–39	301 (1.1%)	449 (1.5%)	193 (0.6%)	118 (1.4%)	1061 (1.1%)
40–49	1142 (4.2%)	1543 (5.3%)	907 (2.8%)	586 (6.9%)	4178 (4.3%)
50–59	3666 (13.4%)	4987 (17.0%)	3781 (11.6%)	1727 (20.4%)	14,161 (14.5%)
60–69	6952 (25.4%)	8426 (28.7%)	8706 (26.7%)	2350 (27.8%)	26,434 (27.1%)
70–79	9539 (34.9%)	9447 (32.2%)	12,276 (37.7%)	2555 (30.2%)	33,817 (34.6%)
** ≥ **80	5724 (20.9%)	4476 (15.3%)	6709 (20.6%)	1116 (13.2%)	18,025 (18.5%)
**Sex**					
Male	11,763 (43.1%)	18,143 (61.9%)	19,280 (59.2%)	4497 (53.2%)	53,683 (55.0%)
Female	15,561 (56.9%)	11,185 (38.1%)	13,292 (40.8%)	3955 (46.8%)	43,993 (45.0%)
**SEER stage**					
Localized	5097 (18.7%)	6650 (22.7%)	8319 (25.5%)	2779 (32.9%)	22,845 (23.4%)
Regional	9265 (33.9%)	7870 (26.8%)	13,945 (42.8%)	3831 (45.3%)	34,911 (35.7%)
Distant	9746 (35.7%)	9796 (33.4%)	4097 (12.6%)	830 (9.8%)	24,469 (25.1%)
Unknown	3216 (11.8%)	5012 (17.1%)	6211 (19.1%)	1012 (12.0%)	15,451 (15.8%)
**First course of treatment**					
Surgical first course of treatment	12,000 (43.9%)	6739 (23.0%)	13,405 (41.2%)	5494 (65.0%)	37,638 (38.5%)
Non-surgical first course of treatment	3877 (14.2%)	6723 (22.9%)	2658 (8.2%)	450 (5.3%)	13,708 (14.0%)
No active anti-cancer treatment	10,807 (39.6%)	15,471 (52.8%)	16,135 (49.5%)	2445 (28.9%)	44,858 (45.9%)
Unknown	640 (2.3%)	395 (1.3%)	374 (1.1%)	63 (0.7%)	1472 (1.5%)

**Figure 1:**
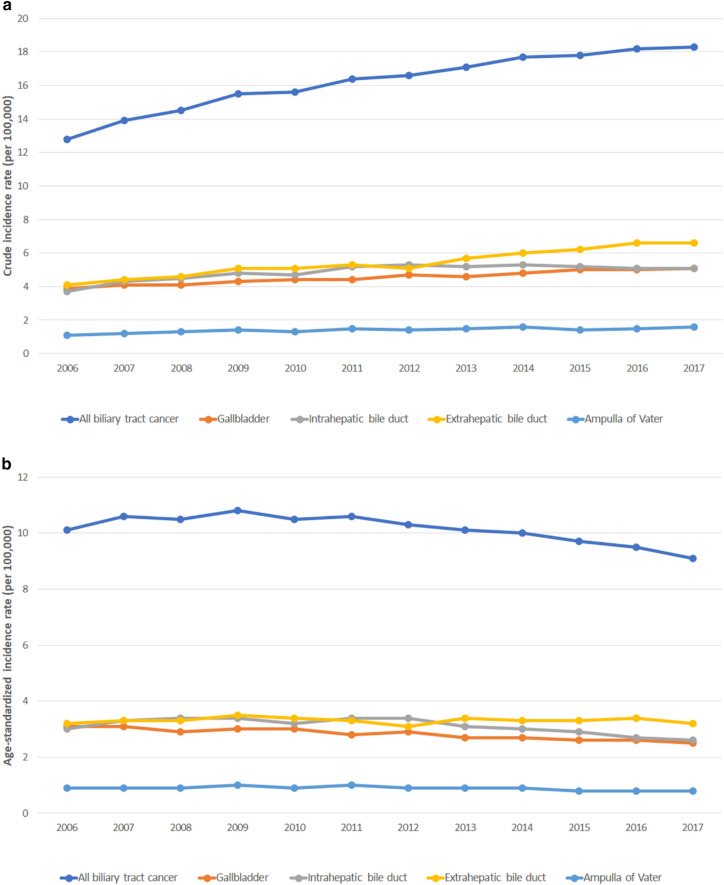
Incidence of biliary tract cancer according to tumor location. (**a**) Crude incidence rate. (**b**) Age-standardized incidence rate.

From 2006 to 2017, the CR of all BTC increased from 12.8 to 18.3 per 100,000 population (Fig. 1a). The ASR decreased in all BTC (10.1–9.1), GB cancer (3.1–2.5), and intrahepatic BD cancer (3.0–2.6), while that of extrahepatic BD cancer (3.2–3.2) and AoV cancer (0.9–0.8) remained stable (Fig. 1b).

### Changes in SEER stage distribution and treatment pattern of biliary tract cancer

**Figure 2:**
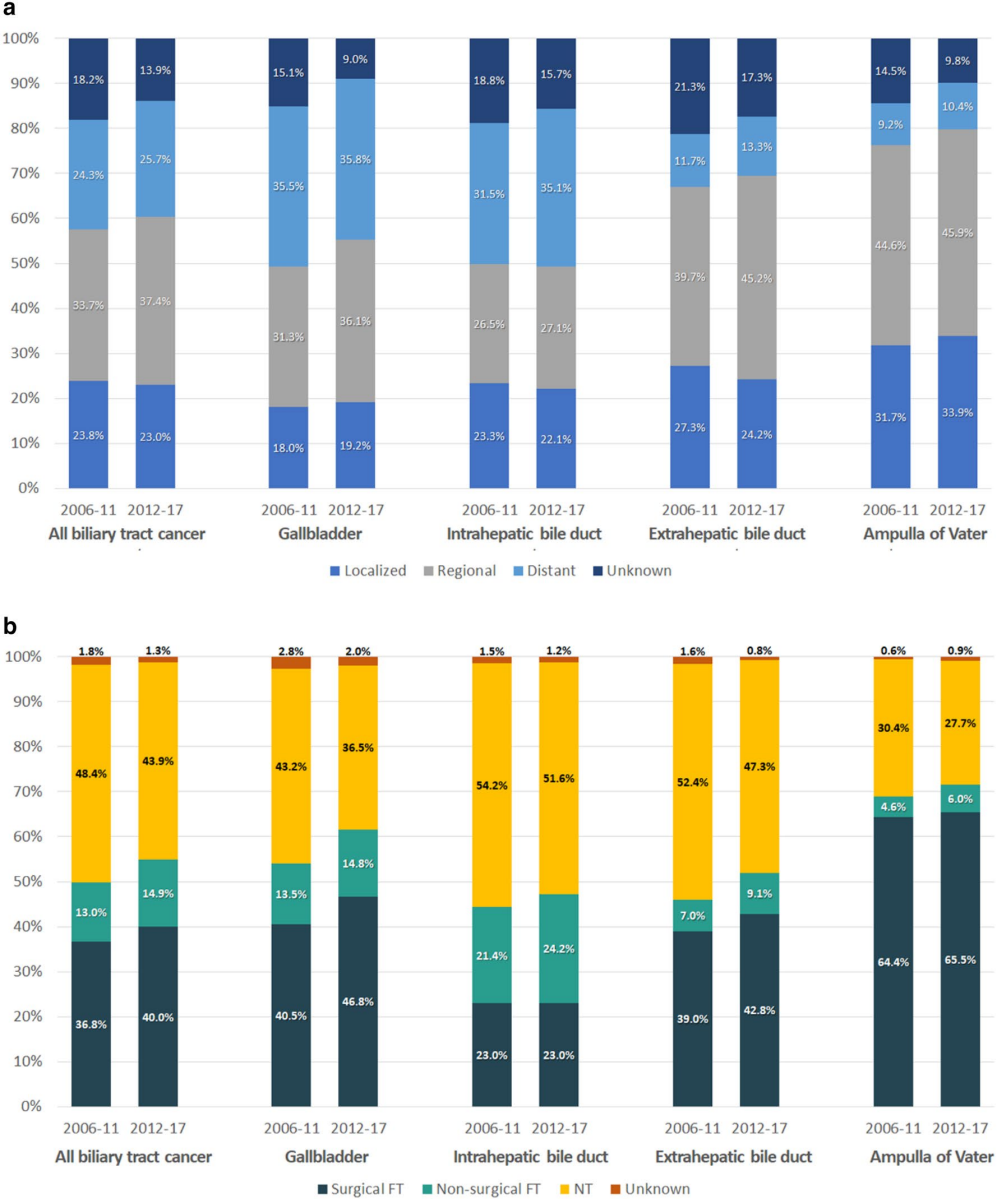
Change of SEER stage distribution and treatment pattern according to tumor location. (**a**) SEER stage distribution according to tumor location. (**b**) Treatment pattern according to tumor location.

The proportion of localized and regional stages, which include potentially resectable disease, was the highest in AoV cancer (78.2%), followed by extrahepatic BD (68.3%), GB (52.6%), and intrahepatic BD cancers (49.5%; Table 1). Comparison of periods I (n = 43,981) and II (n = 53,695) showed that the proportion of localized and regional stages increased in GB (49.4–55.2%, p < 0.001), extrahepatic BD (67.0–69.4%, p < 0.001), and AoV cancers (76.3–79.8%), except for intrahepatic BD cancer (49.8–49.3%, p = 0.384; Fig. 2a).

Of all SEER stages, the proportion of patients receiving surgical first course of treatment was the highest in AoV cancer (65.0%), followed by GB (43.9%), extrahepatic BD (41.2%), and intrahepatic BD cancers (23.0%, p < 0.001; Table 1). From period I to II, the proportion of patients receiving surgical first course of treatment increased for BTC (p < 0.001), GB cancer (p < 0.001), and extrahepatic BD cancer (p < 0.001), but not for intrahepatic BD (p = 0.969) and AoV cancers (p = 0.297; Fig. 2b).

To investigate the factors affecting the risk of receiving no active anti-cancer treatment, patients with localized and regional SEER stage were further analyzed after excluding distant and unstaged patients; intra- and extrahepatic BD cancer had a 71.0% (95% confidence interval [CI] 64.4–77.8, p < 0.001) and 53.8% (95% CI 47.8–60.0, p < 0.001) increased risk of no active anti-cancer treatment, while AoV cancer had a 9.1% lower risk of no active anti-cancer treatment (95% CI 84.8–97.4, p = 0.007) compared to GB cancer after adjusting for period, age group, and sex. Of each tumor location, the risk of no active anti-cancer treatment significantly decreased in period II in GB (42.1%), extrahepatic BD (26.4%), intrahepatic BD cancers (21.7%) and all BTC (18.7%) after adjusting for age and sex. Patients in the older age group had a significantly higher risk of receiving no active anti-cancer treatment after adjusting for period and sex (p < 0.001; Table 2).

**Table 2:** Risk of receiving no active anti-cancer treatment within the first four months after the diagnosis of localized and regional biliary tract cancer according to the tumor location.

	Gallbladder		Intrahepatic bile duct		Extrahepatic bile duct		Ampulla of Vater		All biliary tract cancer	
	Risk ratio (95% CI)	P-value	Risk ratio (95% CI)	P-value	Risk ratio (95% CI)	P-value	Risk ratio (95% CI)	P-value	Risk ratio (95% CI)	P-value
**Period**										
2006–2011	1		1		1		1		1	
2012–2017	0.579 (0.460–0.728)	< 0.001	0.783 (0.699–0.878)	< 0.001	0.736 (0.640–0.845)	< 0.001	0.873 (0.628–1.213)	0.419	0.819 (0.804–0.833)	< 0.001
**Age**										
00–59	1		1		1		1		1	
60–69	1.413 (1.192–1.674)	< 0.001	1.237 (1.130–1.354)	< 0.001	1.303 (1.171–1.450)	< 0.001	1.861 (1.402–2.469)	< 0.001	1.313 (1.250–1.380)	< 0.001
70–79	2.579 (2.214–3.003)	< 0.001	2.010 (1.856–2.177)	< 0.001	2.361 (2.146–2.599)	< 0.001	4.562 (3.563–5.841)	< 0.001	2.435 (2.332–2.543)	< 0.001
≥ 80	4.785 (4.124–5.553)	< 0.001	2.925 (2.708–3.159)	< 0.001	3.890 (3.544–4.270)	< 0.001	11.114 (8.786–14.058)	< 0.001	4.416 (4.234–4.605)	< 0.001
**Sex**										
Male	1		1		1		1		1	
Female	1.030 (0.958–1.108)	0.417	1.033 (0.996–1.071)	0.084	1.069 (1.034–1.106)	< 0.001	1.020 (0.925–1.125)	0.687	1.009 (0.991–1.027)	0.313

### Overall prognosis of patients with biliary tract cancer

**Figure 3:**
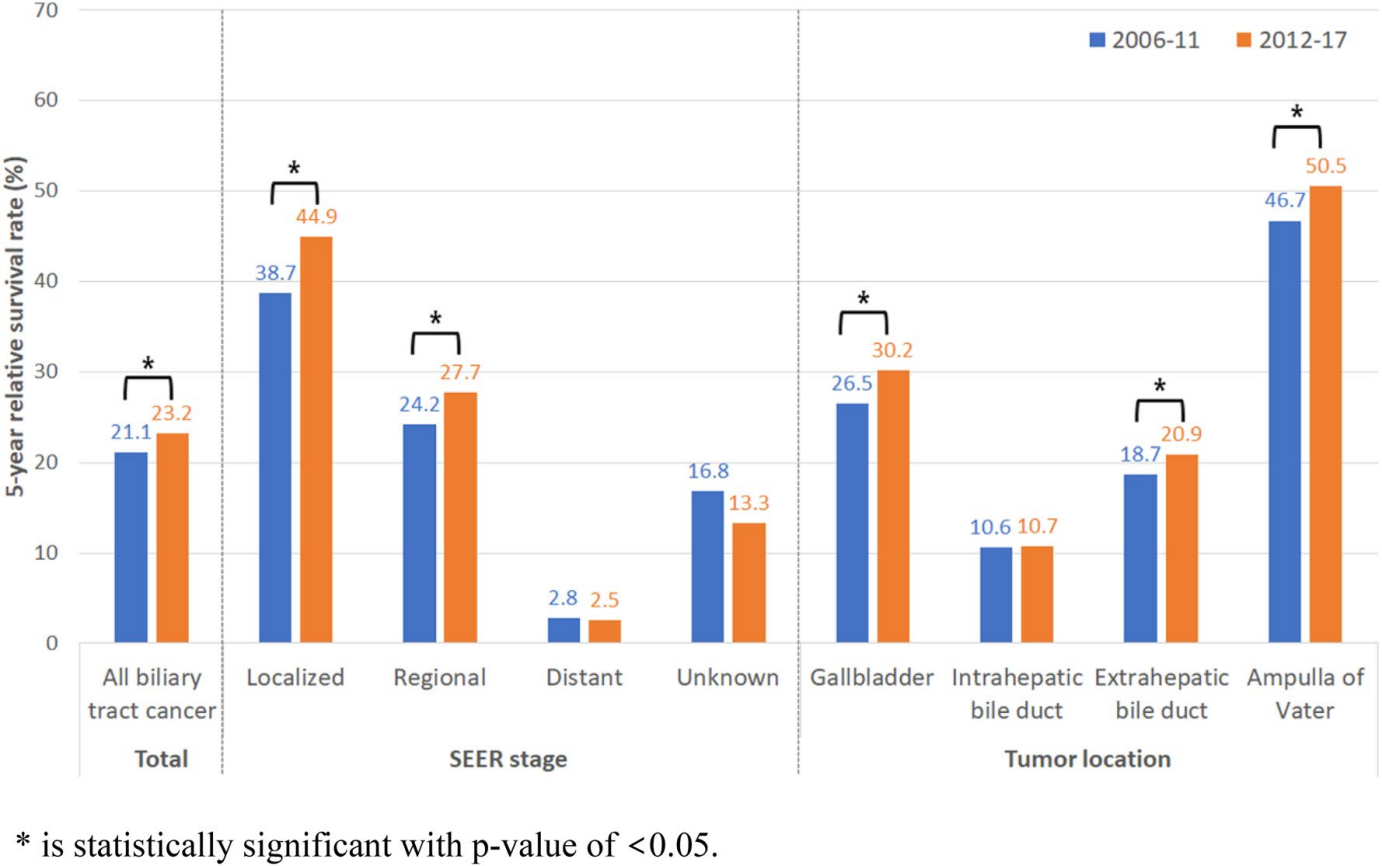
Five-year relative survival rate of biliary tract cancer according to tumor location and SEER stage.

During the study period, the overall 5-year relative survival rate (5YRS) was the highest in AoV cancer (48.5%), followed by GB (28.5%), extrahepatic BD (19.9%), and intrahepatic BD cancers (10.8%). From period I to II, the 5YRS of BTC significantly increased in the overall (p < 0.001), localized (p = 0.012), and regional stages (p < 0.001). According to tumor location, the overall 5YRS significantly increased in period II in GB (p < 0.001), extrahepatic BD (p < 0.001), and AoV cancers (p < 0.001), but not in intrahepatic BD cancer (p = 0.074; Fig. 3).

### Treatment pattern and prognosis of biliary tract cancer according to tumor location

Due to the minimal impact of treatment pattern on the prognosis at the distant stage, further results are described focusing on the localized and regional SEER stage disease with available information on treatment.

#### Gallbladder cancer

**Figure 4:**
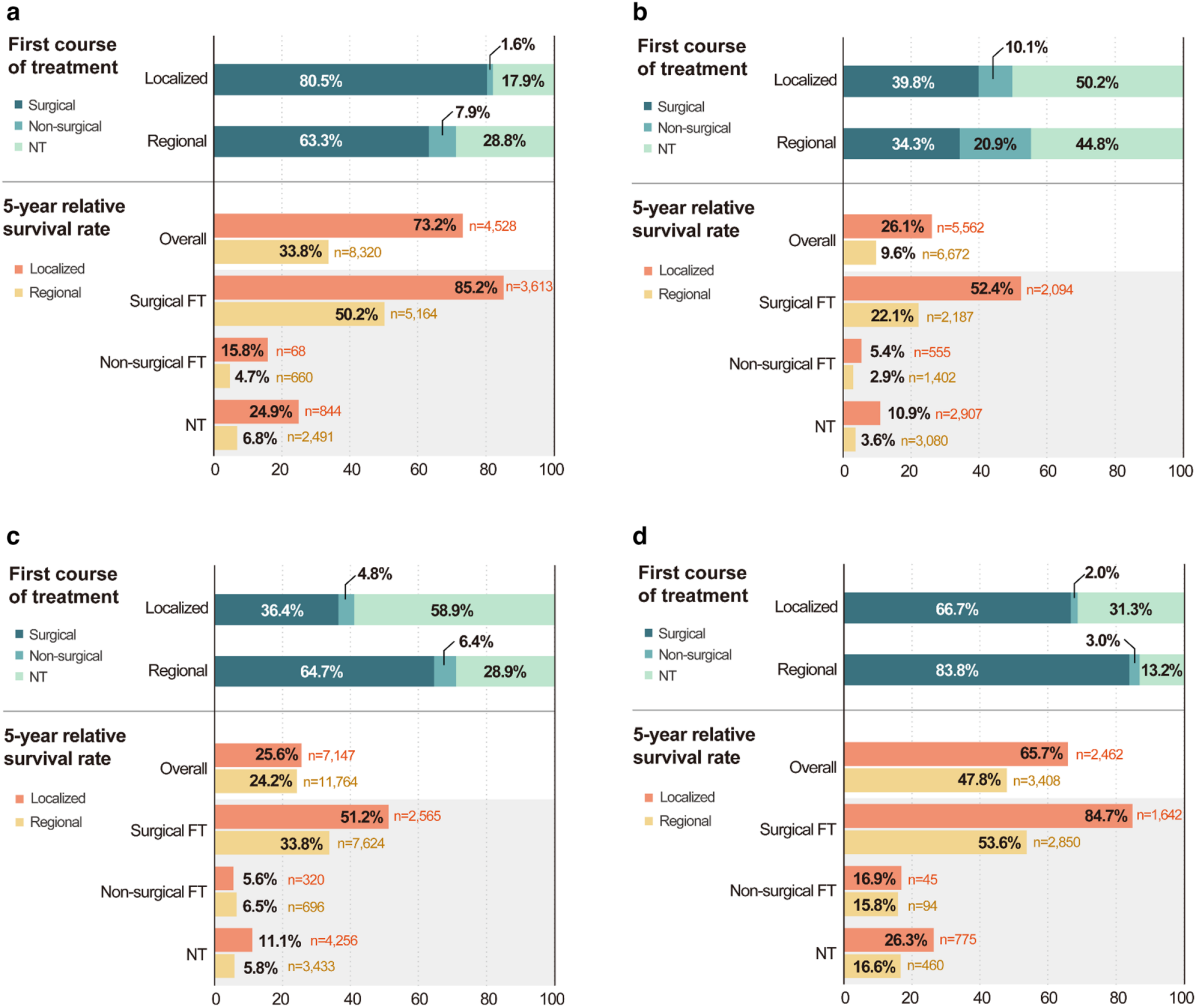
Treatment pattern and 5-year relative survival rate of localized and regional SEER stage biliary tract cancer according to tumor location. *NT no active anti-cancer treatment, *FT* first course of treatment. (**a**) Gallbladder cancer. (**b**) Intrahepatic bile duct cancer. (**c**) Extrahepatic bile duct cancer. (**d**) Ampulla of Vater cancer.

Among 14,352 patients with localized and regional stage GB cancer, 80.5% of the localized stage and 63.3% of the regional stage patients received surgical first course of treatment (Fig. 4a). From period I to II, the proportion of patients undergoing surgical first course of treatment increased and that of patients receiving no active anti-cancer treatment decreased in both the localized (77.0–83.2% [surgical first course of treatment]; 20.9–15.6% [no active anti-cancer treatment]; p < 0.001) and regional stages (58.1–67.0% [surgical first course of treatment]; 33.4–25.4% [no active anti-cancer treatment]; p < 0.001; Supplementary Fig. 1a). The overall 5YRS of the localized and regional stage GB cancer between 2006 and 2017 was 73.2% (localized) and 33.8% (regional), respectively, which was significantly improved by 6.4% in the regional stage in period II (Fig. 4a, Supplementary Fig. 1b).

#### Intrahepatic bile duct cancer

Among 14,510 patients with localized and regional stage intrahepatic BD cancer, 39.8% of the localized stage and 34.3% of the regional stage patients received surgical first course of treatment (Fig. 4b). From period I to II, the proportion of patients undergoing surgical first course of treatment increased and that of patients receiving no active anti-cancer treatment decreased in the localized stage (36.3–42.9% [surgical first course of treatment]; 52.6–48.0% [no active anti-cancer treatment]; p < 0.001; Supplementary Fig. 2a). The overall 5YRS of the localized and regional stage intrahepatic BD cancer between 2006 and 2017 was 26.1% (localized) and 9.6% (regional), respectively, which significantly improved by 5.0% in the localized stage in period II (Fig. 4b, Supplementary Fig. 2b).

#### Extrahepatic bile duct cancer

Among 22,244 patients with localized and regional stage extrahepatic BD cancer, 36.4% of the localized stage and 64.7% of the regional stage patients received surgical first course of treatment (Fig. 4c). From period I to II, the proportion of patients undergoing surgical first course of treatment increased and that of patients receiving no active anti-cancer treatment decreased in both the localized (34.2–38.2% [surgical first course of treatment]; 60.9–57.1% [no active anti-cancer treatment]; p = 0.001) and regional stages (61.3–67.0% [surgical first course of treatment]; 32.6–26.4% [no active anti-cancer treatment]; p < 0.001; Supplementary Fig. 3a). The overall 5YRS of the localized and regional stage extrahepatic BD cancer between 2006 and 2017 was 25.6% (localized) and 24.2% (regional), respectively, which significantly improved by 2.4% in the regional stage in period II (Fig. 4c, Supplementary Fig. 3b).

#### Ampulla of Vater cancer

Among 6,604 patients with localized and regional stage AoV cancer, 66.7% of the localized stage and 83.8% of the regional stage patients received surgical first course of treatment (Fig. 4d). From period I to II, the proportion of patients undergoing surgical first course of treatment increased and that of patients undergoing no active anti-cancer treatment decreased in the localized stage (64.5–68.4% [surgical first course of treatment]; 34.2–29.0% [no active anti-cancer treatment]; p = 0.002; Supplementary Fig. 4a). The 5YRS of the localized and regional stage AoV cancer between 2006 and 2017 was 65.7% (localized) and 47.8% (regional), respectively, without significant changes over time (Fig. 4d, Supplementary Fig. 4b).

## Discussion

BTCs in various tumor locations share a number of characteristics, including pathophysiologic traits, use of surgical resection as a mainstay of potentially curative treatment, and dismal prognosis, especially in the advanced stages^13^. However, the results of studies on BTCs, in whole or in part, have been inconsistent regarding the inclusion of various tumor locations. As shown in this study, each tumor location has a different SEER stage distribution and treatment pattern, resulting in distinct survival outcomes. Over time, the proportion of the localized and regional stage disease, including potentially resectable disease, increased in GB, extrahepatic BD, and AoV cancers, which led to increased surgical first course of treatment rate and 5YRS, except for intrahepatic BD cancer.

In this study, the age-standardized incidence rate of all four tumor locations were higher than that of the SEER database, especially in GB (2.5 [Korea] vs. 1.6 [SEER] per 100,000), intrahepatic BD (2.6 [Korea] vs. 1.3 [SEER]), and extrahepatic BD (3.2 [Korea] vs. 1.2 [SEER]) cancers^22^. Both databases revealed male predominance in intra- and extrahepatic BD cancer, and female predominance in GB cancer^22^. However, the incidence trends of intrahepatic BD cancer decreased and extrahepatic BD cancer remained stable in Korea, while intra- and extrahepatic BD cancer had increasing trends in the SEER database. The increasing trend of intrahepatic BD cancer in the United States have been continuously reported^9,22–24^.

Despite advances in adjuvant chemotherapy and radiation therapy^1,25^, the overall survival outcome of BTC has not improved dramatically. The overall 5YRS of BTC based on population-based registries was reported to be lower than 5% in Western countries^24,26^ and 20–30% in Eastern countries^27,28^. This discrepancy results from the fact that the registry data include all stages of disease originating from various tumor locations with or without cancer-directed treatment. In this study, the 5YRS for each tumor location with the localized and regional stage disease receiving surgical first course of treatment, which may approximate resectable disease with or without lymph node metastasis, was in concordance with that of clinical studies after R0 resection for American Joint Committee on Cancer (AJCC) stage I–II patients^29–32^. For each tumor location and SEER stage, surgical first course of treatment resulted in better prognosis than non-surgical first course of treatment or no active anti-cancer treatment. Consequently, a higher proportion of no active anti-cancer treatment was related to a larger difference in 5YRS between the overall (regardless of first course of treatment) and surgical first course of treatment groups. In this study, the overall 5YRS for each stage and tumor location was 6–27% lower than that after surgical first course of treatment. In particular, although the 5YRS of localized stage intra- and extrahepatic BD cancers after surgical first course of treatment exceeded 50%, the overall prognosis of these cancers was approximately half of that of the surgical first course of treatment group since more than 50% of the patients received no active anti-cancer treatment. Therefore, it is crucial to reduce the proportion of patients who do not receive cancer-directed treatment to improve the overall survival rate of BTC in the population. As shown in this study, patients in their 60 s and 70 s had a 1.2–1.9-fold and 2.0–4.6-fold higher risk of receiving no active anti-cancer treatment, respectively, compared to those under the age of 60 years. Considering the aging society and that the median age of BTC patients is above 70 years, treatment guidelines adjusted for elderly patients are required to broaden the scope of patients actively receiving cancer-directed treatment^15^. On the other hand, based on social norms, it was assumed that female patients would be more likely to receive no active anti-cancer treatment; however, the likelihood of receiving no active anti-cancer treatment was increased in female patients only in extrahepatic BD cancer after adjusting for period and age in the localized and regional stage disease.

This study revealed that the no active anti-cancer treatment rate of BTC was 45.9% in all SEER stages and 35.9% in the localized and regional stage. Among the localized and regional stage BTC, the no active anti-cancer treatment rate was the highest in intrahepatic BD cancer (47.2%), followed by extrahepatic BD (40.1%), GB (25.0%), and AoV cancers (20.8%). General perception of the poor prognosis of bile duct cancer and the fatality of liver surgery may have led to the misconception that the benefits of surgical treatment might be less than that for GB or AoV cancers and affected the patients’ decision-making.

Noticeably, the proportion of no active anti-cancer treatment was higher in the localized stage than in the regional stage in extrahepatic BD and AoV cancers. The majority of these cancers present with obstructive jaundice; therefore, biliary drainage is required before surgery or chemoradiotherapy, which can be maintained for several weeks to months. In particular, planned liver resection in patients with perihilar cholangiocarcinoma require stricter biliary decompression. Consequently, prolonged stenting for biliary decompression may lead to overestimation of the no active anti-cancer treatment rate since the cancer registry does not report on the treatment information after the first four months of the initial diagnosis. The microscopic verification rate of localized stage intra- and extrahepatic BD cancer was 23–32% lower than that of GB and AoV cancer. Therefore, the localized stage no active anti-cancer treatment group of intra- and extrahepatic BD cancer may have included patients with equivocal lymph node involvement who could have been classified as regional stage if they underwent surgery. In addition, the high rate of no active anti-cancer treatment and the resulting poor prognosis of intrahepatic BD cancer can be explained by the definition of the SEER stage: localized intrahepatic BD cancer includes a wide spectrum of diseases ranging between AJCC stage I and stage IIIA^33^.

This study has several limitations. First, the inability to match the SEER stage with the AJCC TNM stage remains one of the main drawbacks of cancer registry studies, which makes it difficult to extrapolate the clinical implications of the results. Second, the lack of information on cancer-directed treatment administered later than four months after the initial diagnosis may have overestimated the proportion of patients receiving no active anti-cancer treatment, especially for those who had prolonged preoperative biliary drainage. Third, in addition to the lack of precise pathological staging, limited information on detailed treatment including operation name, chemotherapeutic agent, or immunotherapy made it impossible to recognize the intent (curative or palliative) or outcomes of various treatment modalities. Future studies in collaboration with KCCR and National Health Insurance Claim Data would improve the clarity in treatment outcomes analysis and defining a group of patients who need to be monitored closely to reduce the proportion of patients with no active anti-cancer treatment, improving the overall survival outcomes of BTC.

In summary, BTC has distinct epidemiological and clinical characteristics, treatment patterns, and prognosis according to tumor location. For each tumor location, elderly patients had a higher risk of receiving no active anti-cancer treatment. Despite favorable prognosis after surgical first course of treatment, localized stages of intra- and extrahepatic BD cancer had a high proportion of patients receiving no active anti-cancer treatment, resulting in a poor overall 5YRS in the population. Therefore, BTC statistics should be separately reported according to the tumor location to provide information on the condition’s unique features. In addition, addressing the survival gain in actively treated BTC patients should be emphasized. Efforts should be made to reduce the proportion of patients not receiving cancer-directed treatment, especially for those in their 60 s and 70 s, to improve overall survival outcomes of BTC in the population.

## Supplementary Information

Supplementary Figures.

## Data Availability

The datasets generated during and/or analysed during the current study are available from the corresponding author on reasonable request.
