# Supplementary material for: Distinct prognosis of biliary tract cancer according to tumor location, stage, and treatment: a population-based study
Source: Sci Rep. 2022 Jun 17;12:10206. doi: 10.1038/s41598-022-13605-3 (PMC9205970; doi:10.1038/s41598-022-13605-3)

Original article

Distinct prognosis of biliary tract cancer according to tumor location, stage, and treatment: a population-based study

Mee Joo Kang[1, 2], Jiwon Lim[2], Sung-Sik Han[1], Hyeong Min Park[1], Sun-Whe Kim[1], Woo Jin Lee[1], Sang Myung Woo[1], Tae Hyun Kim[1], Young-Joo Won[2, 3, *], and Sang-Jae Park[1, *]

[1] Center for Liver and Pancreatobiliary Cancer, National Cancer Center, Goyang, Korea

[2] Division of Cancer Registration and Surveillance, National Cancer Center, Goyang, Korea

[3] Department of Cancer Control & Population Health, Graduate School of Cancer Science and Policy, National Cancer Center, Goyang, Korea

***Correspondence to**:

Sang-Jae Park, M.D., Ph.D.

Center for Liver and Pancreatobiliary Cancer, National Cancer Center

323 Ilsan-ro, Ilsandong-gu, Goyang-si Gyeonggi-do, 10408, Republic of Korea

Tel: 82-31-920-1640 E-mail: spark@ncc.re.kr

Young-Joo Won, Ph.D.

Korea Central Cancer Registry, National Cancer Center

323 Ilsan-ro, Ilsandong-gu, Goyang-si Gyeonggi-do, 10408, Republic of Korea

Tel: 82-31-920-2015 E-mail: astra67@ncc.re.kr

Supplementary figures

Supplementary figure 1. Gallbladder cancer

Supple Figure 1a. Treatment pattern according to SEER stage and time period

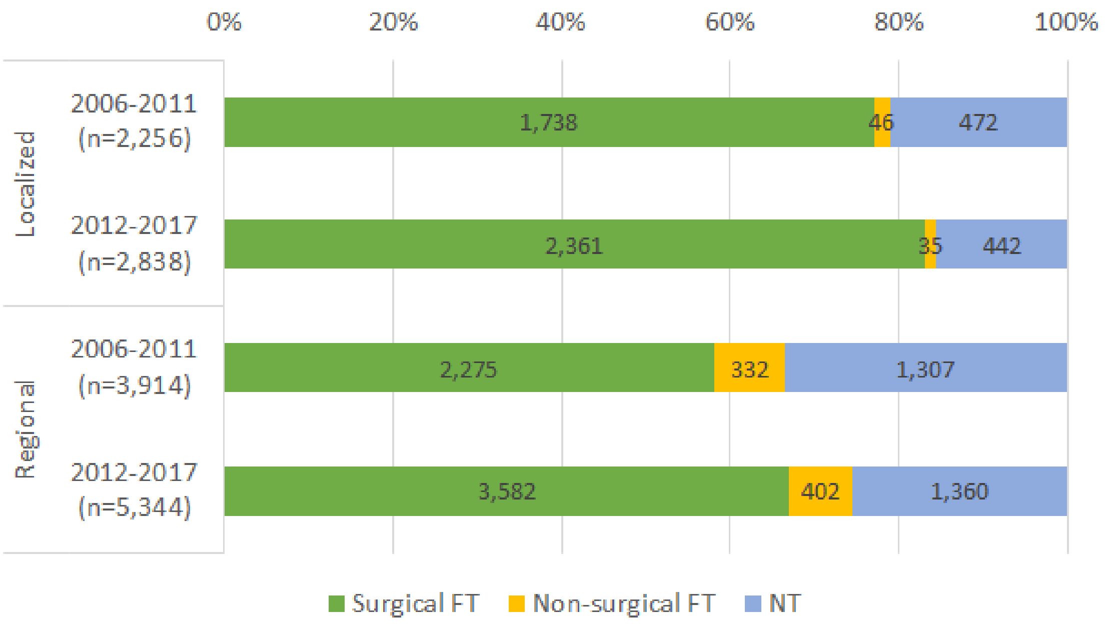

Supple Figure 1b. Five-year relative survival rate according to SEER stage and first course of treatment

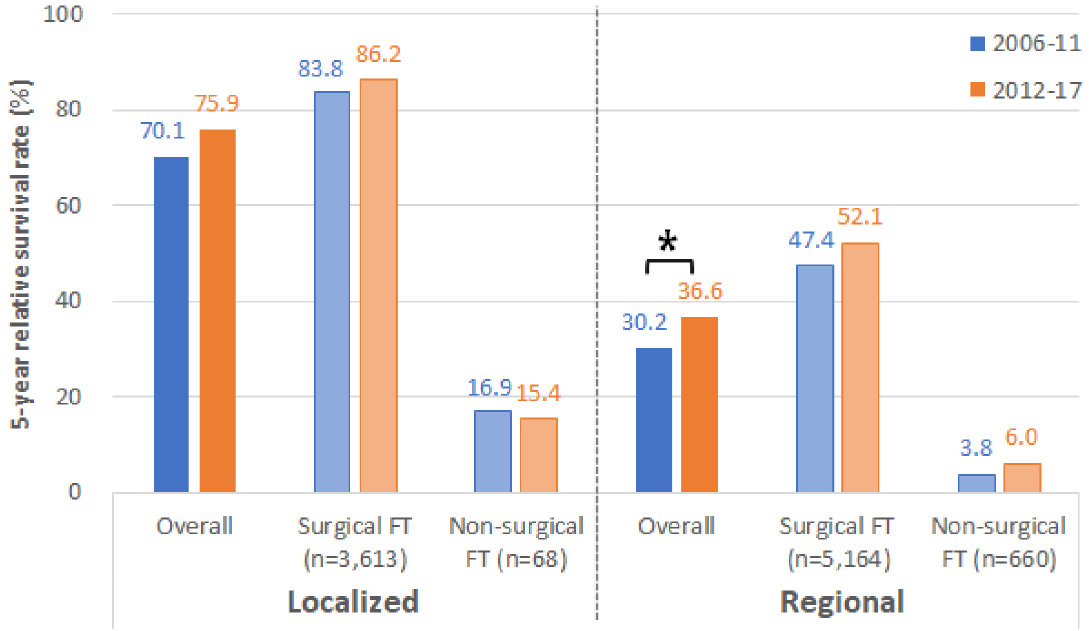

* is statistically significant with p-value of <0.05.

Supplementary figure 2. Intrahepatic bile duct cancer

Supple Figure 2a. Treatment pattern according to SEER stage and time period

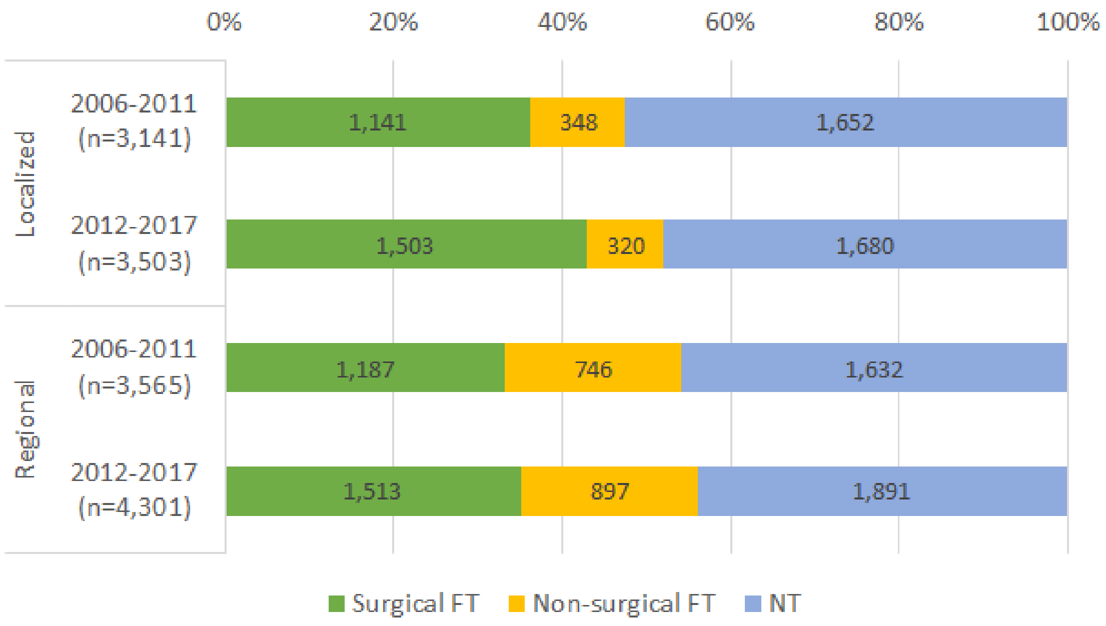

Supple Figure 2b. Five-year relative survival rate according to SEER stage and first course of treatment

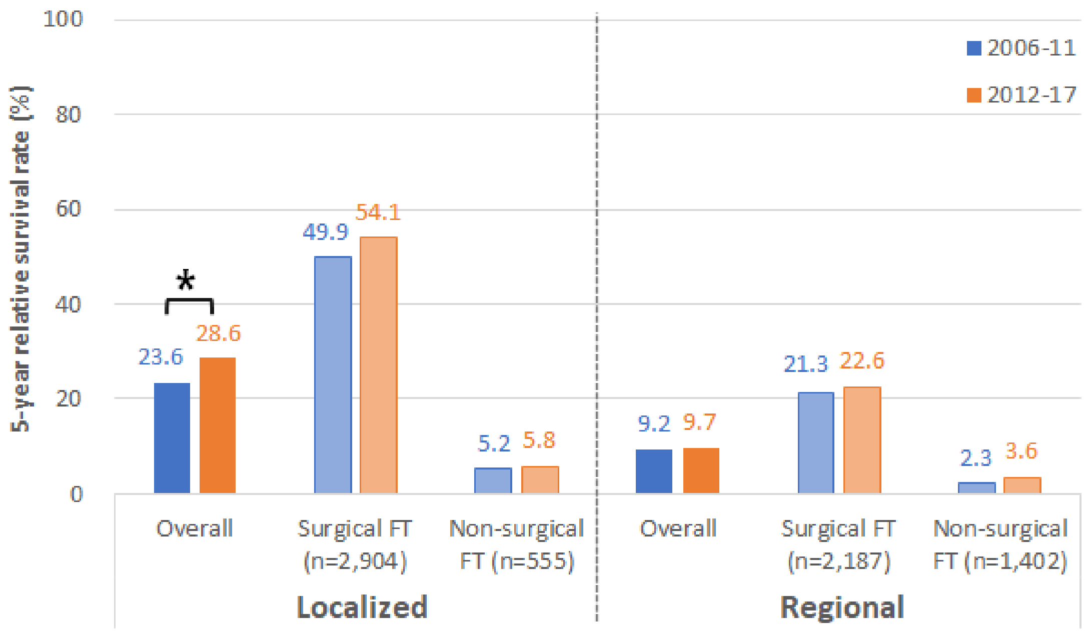

* is statistically significant with p-value of <0.05.

Supplementary figure 3. Extrahepatic bile duct cancer

Supple Figure 3a. Treatment pattern according to SEER stage and time period

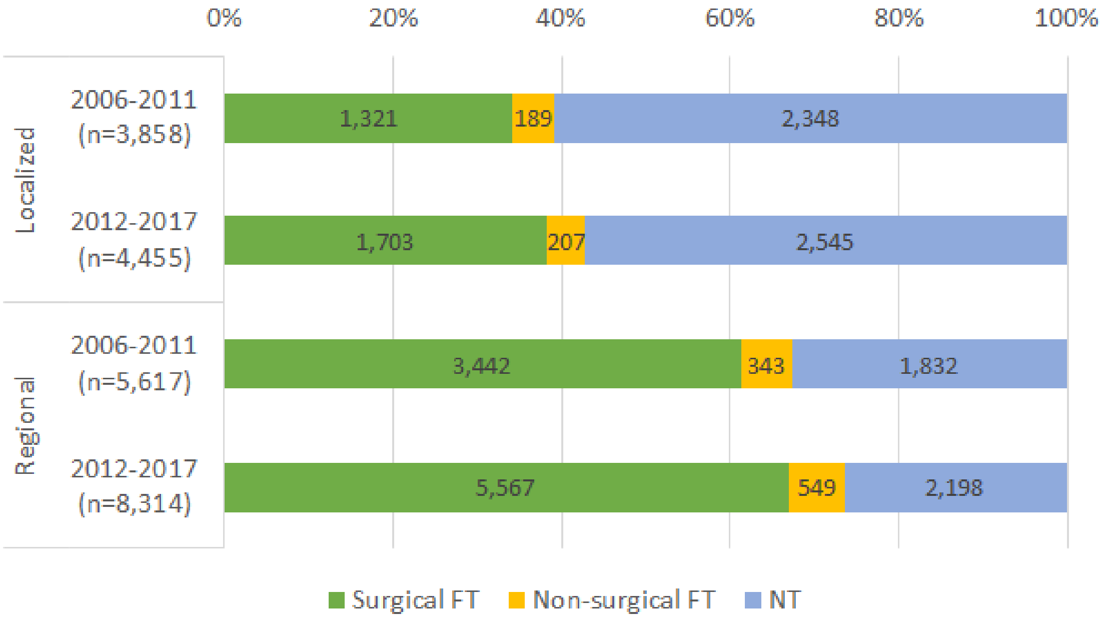

Supple Figure 3b. Five-year relative survival rate according to SEER stage and first course of treatment

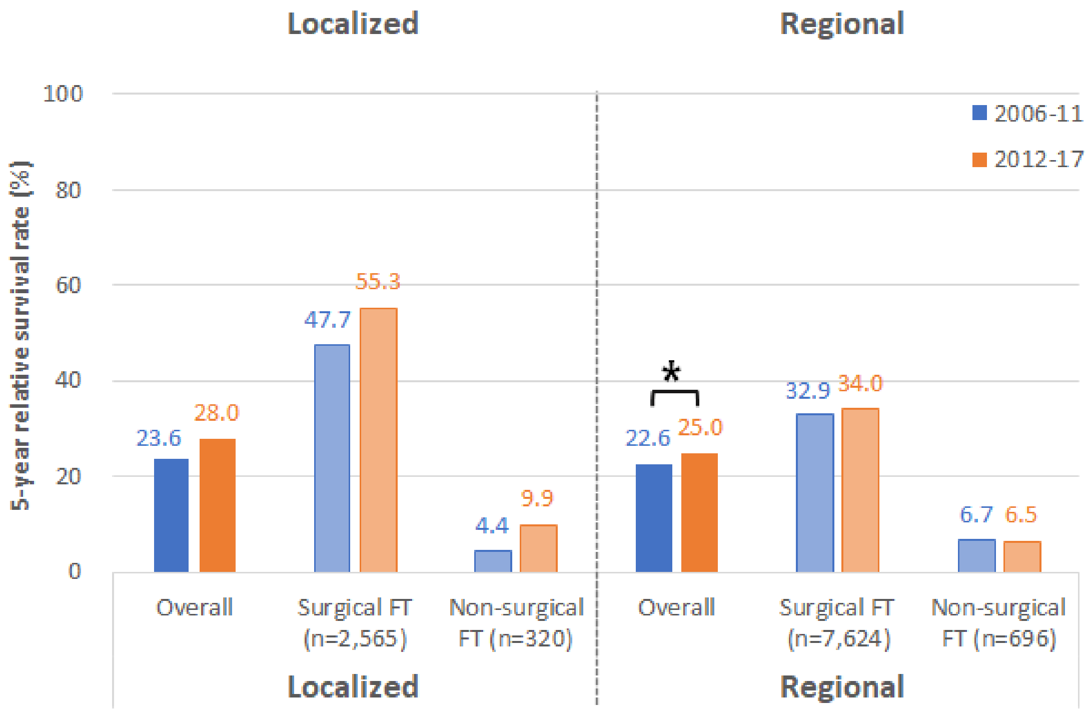

* is statistically significant with p-value of <0.05.

Supplementary figure 4. Ampulla of Vater cancer

Supple Figure 4a. Treatment pattern according to SEER stage and time period

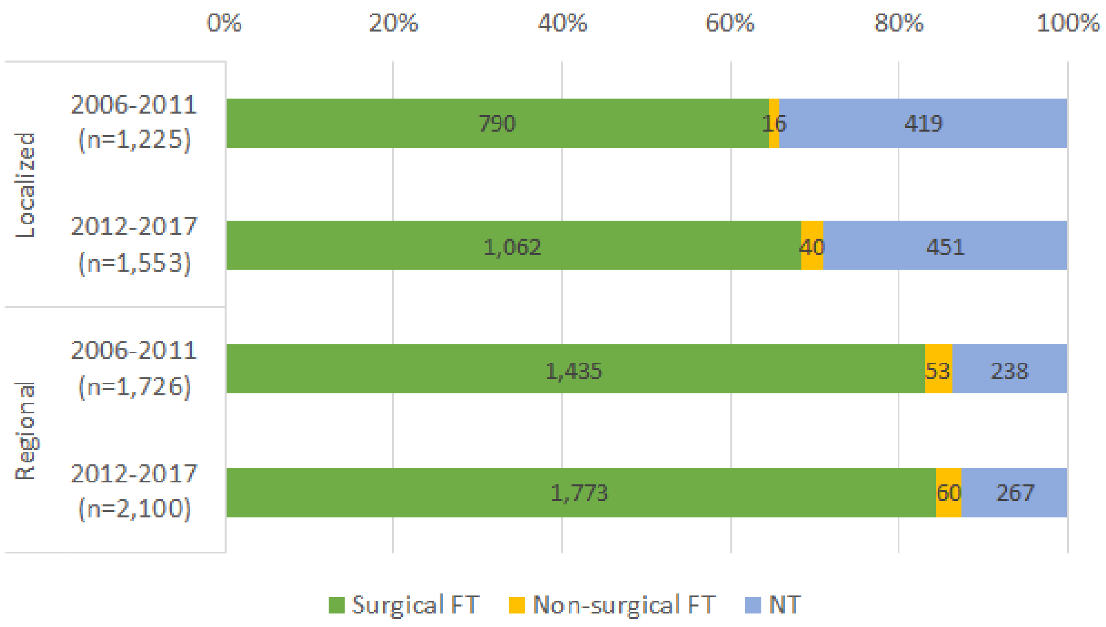

Supple Figure 4b. Five-year relative survival rate according to SEER stage and first course of treatment

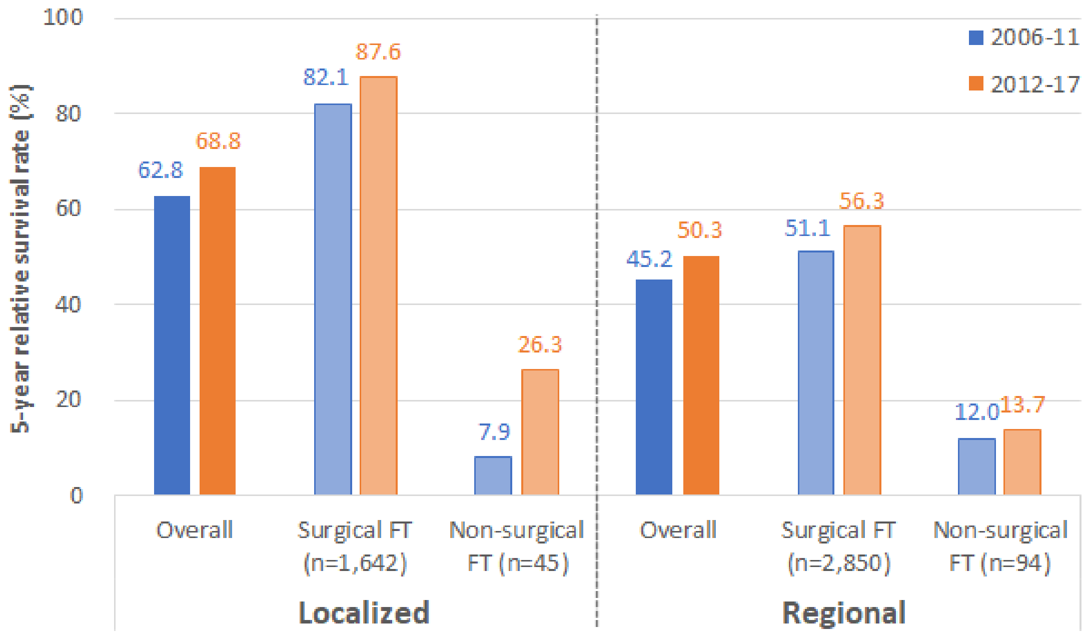

Supplement: Supplementary file 1 — Supplementary Figures. [file 41598_2022_13605_MOESM1_ESM.pdf]
